# Outcomes of 10 mg Rivaroxaban in Nonvalvular Atrial Fibrillation Patients With CrCl ≥ 50 mL/min: A Retrospective Cohort Study

**DOI:** 10.1155/cdr/7021330

**Published:** 2025-06-26

**Authors:** Qing Yan, Yide Yuan, Jiaqi Liang, Yuyang Zhao, Yuan Li, Jiali Fan, Jiahong Xue, Qiangsun Zheng

**Affiliations:** ^1^Department of Cardiovascular Medicine, The Second Affiliated Hospital of Xi'an Jiaotong University, Xi'an, Shaanxi, China; ^2^Department of Cardiovascular Medicine, Xidian Group Hospital, Xi'an, Shaanxi, China; ^3^Department of Oncology and Cardiology, Xinjiang Medical University Affiliated Cancer Hospital, Urumqi, Xinjiang, China

**Keywords:** bleeding, ischemic stroke, nonvalvular atrial fibrillation, rivaroxaban, systemic embolism

## Abstract

**Background:** The standard oral dose of rivaroxaban for nonvalvular atrial fibrillation (NVAF) patients with a creatinine clearance rate (CrCl) ≥ 50 m*L*/min is 20 mg/day in Europe and 15 mg/day in Japan. In the real world, low-dosing (10 mg/day) rivaroxaban has been widely used in clinical practice in China due to bleeding concerns. However, the impact of low-dose rivaroxaban on clinical outcomes remains uncertain.

**Methods:** This retrospective study included NVAF patients with CrCl ≥ 50 mL/min treated at the Second Affiliated Hospital of Xi'an Jiaotong University from January 2017 to June 2022. Patients were divided into two groups: standard-dose (15 or 20 mg/day) and low-dose (10 mg/day). Inverse probability of treatment weighting (IPTW) was used to balance baseline characteristics. The risk of ischemic stroke (IS)+systemic embolism (SE) and bleeding was compared between the two groups by survival analysis.

**Results:** A total of 1455 patients (mean age: 66.98 ± 11.16 years; 55.95% female) were included. In the low-dose group (*n* = 1176), 78 (6.63%) experienced IS/SE and 68 (5.78%) had bleeding. In the standard-dose group (*n* = 279), 13 (4.66%) experienced IS/SE and 18 (6.45%) had bleeding. Cox regression suggested that compared to the standard-dose group, patients in the low-dose group did not show a significantly different risk of IS+SE (HR = 1.01, 95% CI: 0.51–1.96, *p* = 0.999) or bleeding (HR = 0.90, 95% CI: 0.49–1.67, *p* = 0.749). Subgroup analysis revealed that for patients with BMI < 24 kg/m^2^, low-dose rivaroxaban reduced bleeding risk (HR = 0.53, 95% CI: 0.29–0.99, *p* = 0.049).

**Conclusion:** For NVAF patients with CrCl ≥ 50 mL/min in China, low-dose rivaroxaban (10 mg/day) is a viable alternative to standard doses in preventing IS/SE. In nonoverweight patients (BMI < 24 kg/m^2^), it offers comparable efficacy with enhanced safety.

## 1. Introduction

Atrial fibrillation (AF) is the most common cardiac arrhythmia, with a prevalence of approximately 1.8% in Chinese adults [1]. It increases the risk of ischemic stroke (IS) fivefold [2], and oral anticoagulants have been shown to effectively reduce this risk [3, 4]. For decades, vitamin K antagonists, such as warfarin, were the primary drugs for preventing systemic embolism (SE) in nonvalvular atrial fibrillation (NVAF) patients [4, 5]. However, with the advent of novel oral anticoagulants (NOACs), such as rivaroxaban, studies have demonstrated that these agents are at least as effective as warfarin in preventing strokes and may also reduce the risk of bleeding [6, 7].

Based on the results of the ROCKET AF study in 2011 [8, 9], international authoritative guidelines for AF diagnosis and treatment recommend that NVAF patients at high risk of stroke can take rivaroxaban orally to prevent IS, and the recommended dose is 20 mg/day (15 mg/day for patients with a CrCl < 50 mL/min) [10, 11]. Subsequently, the J-ROCKET study in Japan found that 15 mg/day (10 mg/day for CrCl < 50 mL/min) was equally effective as warfarin in preventing IS/SE, with a lower incidence of bleeding [12]. Based on these findings, both Taiwan and Japan now permit 15 or 10 mg/day (for CrCl < 50 mL/min) dosing of rivaroxaban for NVAF patients [13–15].

However, many NVAF patients with CrCl ≥ 50 mL/min in real world in China are prescribed 10 mg/day rivaroxaban due to bleeding concerns. Studies from Taiwan and Japan have yielded conflicting results: some suggest that 10 mg/day increases the risk of IS [16, 17] while others find it comparable in effectiveness and safety to standard doses [18, 19]. In this study, we aimed to evaluate the effectiveness and safety outcomes of 10 mg rivaroxaban compared to the recommended dose in NVAF patients with CrCl ≥ 50 mL/min in mainland China.

## 2. Methods

### 2.1. Study Design and Population

**Figure 1:**
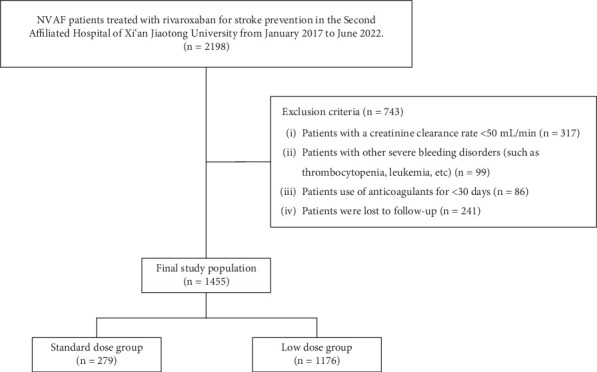
Flowchart of the study population.

This was a retrospective cohort study conducted at the Second Affiliated Hospital of Xi'an Jiaotong University. The inclusion criteria were as follows: (1) NVAF patients with a definite clinical diagnosis and (2) patients treated with rivaroxaban for stroke prevention. From January 2017 to June 2022, we identified 2198 NVAF patients treated with rivaroxaban from the electronic medical records. In the present study, we employed a stepwise exclusion process with a single-trigger mechanism to select the final study population. And 743 patients who met the exclusion criteria were excluded. In detail, 317 patients with a CrCl < 50 mL/min, 99 patients with other severe bleeding disorders (such as thrombocytopenia and leukemia), 86 patients using anticoagulants for < 30 days, and 241 patients were lost to follow-up. Finally, 1455 patients have constituted the study population (Figure 1).

This study complied with the Declaration of Helsinki and was approved by the Ethics Review Board of the Second Affiliated Hospital of Xi'an Jiaotong University (No. 2022048). Oral or written informed consent for the study was obtained from all participants.

### 2.2. Grouping of Subjects

Among the 1455 patients in the study population, 102 (7.01%) received rivaroxaban at 20 mg/day, 177 (12.17%) received 15 mg/day, and 1176 (80.82%) received 10 mg/day. Patients were further divided into two groups based on the dose of rivaroxaban they received: (1) standard-dose group (*n* = 279; 19.18%)—patients who received daily doses of rivaroxaban according to the ROCKET-AF (20 mg/day) or J-ROCKET (15 mg/day) studies, and (2) low-dose group (*n* = 1176; 80.82%)—patients who received a daily dose of 10 mg of rivaroxaban (Figure 1).

### 2.3. Baseline Data

Demographic parameters and comorbidities were collected from patient medical records. It included gender, age, body mass index (BMI), hypertension, diabetes, hyperlipidemia (including high cholesterol and triglyceride), coronary atherosclerotic heart disease (CAD), AF type, utilization of antiplatelet and antiarrhythmic drugs (AADs), left atrial diameter (LAD), and left ventricular ejection fraction (LVEF). Besides, CHA2DS2-VASc and HAS-BLED scores were calculated to assess the risk of stroke and bleeding, respectively.

### 2.4. Outcome Definition

The primary effectiveness outcome of the study was a composite of IS and SE, while the primary safety outcome encompassed any bleeding, including both major and nonmajor events. To ensure accurate classification and avoid misclassification, all outcomes were based on the primary discharge diagnosis. IS, SE, and bleeding events were confirmed when the primary diagnostic code of hospitalization was supported by concomitant imaging studies, such as computed tomography or magnetic resonance imaging [20]. According to the definitions used in the Japanese J-ROCKET and XAPASS studies [12, 21], IS is defined as a new sudden, focal neurological deficit resulting from a presumed cerebrovascular cause, persisting beyond 24 h and unattributable to another readily identifiable cause. SE is defined as abrupt vascular insufficiency associated with clinical or radiological evidence of arterial occlusion in the absence of other likely mechanisms (e.g., trauma, atherosclerosis, or instrumentation). Major bleeding is defined as clinically overt bleeding associated with any of the following: fatal outcome, involvement of a critical anatomic site (intracranial, spinal, ocular, pericardial, articular, retroperitoneal, or intramuscular with compartment syndrome), > 2 g/dL reduction in hemoglobin concentration, transfusion of > 2 units of whole blood or packed red blood cells, or permanent disability. Nonmajor bleeding is defined as overt bleeding not meeting the criteria for major bleeding (including epistaxis, gingival bleeding, scleral hemorrhage, and other nuisance bleedings). Patients were followed up by telephone or through electronic medical records.

### 2.5. Statistical Analysis

Continuous variables are presented as mean ± standard deviation for normally distributed data or as medians with interquartile ranges (IQRs) for non-normally distributed data. Categorical variables are presented as percentages. Differences in baseline characteristics between groups were compared using the chi-square test for categorical variables and one-way analysis of variance (ANOVA) or the Mann–Whitney *U* test for continuous variables, depending on the distribution. To address potential confounding, we employed inverse probability of treatment weighting (IPTW) to balance baseline characteristics between the standard-dose and low-dose groups. Kaplan–Meier (K–M) survival analysis was used to compare clinical outcomes between the groups, while Cox proportional hazards regression models were employed to evaluate the risk factors associated with effectiveness and safety outcomes. IPTW, K–M survival analysis, and Cox proportional hazards regression were performed using R software (Version 4.2.2). Descriptive statistics and data preprocessing were conducted with SPSS (Version 28.0), and figures were generated using GraphPad Prism (Version 8.0.2). *p* value < 0.05 was considered statistically significant.

## 3. Results

### 3.1. Baseline Characteristics

Among the 1455 NVAF patients in this study (mean age: 66.98 ± 11.16 years, BMI: 23.67 ± 3.14 kg/m^2^, 55.95% female), 814 (55.95%) had paroxysmal AF. During a median follow-up of 734 days (743.5 days in the low-dose group and 705 days in the standard-dose group), 91 (6.25%) individuals experienced IS or SE events, while the probability of any bleeding event was 5.91% (*n* = 86).

The baseline characteristics of patients before IPTW are summarized in Table 1. Patients in the low-dose group were more likely to be older (68.00 ± 10.67 vs. 62.67 ± 12.13; *p* < 0.001), had a higher proportion of persistent AF (46.00% vs. 35.84%; *p* = 0.003), and a greater prevalence of CAD (36.39% vs. 22.94%; *p* = 0.001) compared to those receiving the recommended dose. There were no significant differences in gender, CHA2DS2-VASc score, or HAS-BLED score between the two groups. After adjustment using IPTW, all clinical covariates were well balanced (Table 2).

### 3.2. Clinical Outcomes

During the follow-up period, 78 patients in the low-dose group experienced IS+SE (72 with IS and six with SE) compared to 13 patients in the standard-dose group (11 with IS and two with SE). Additionally, the incidence of any bleeding events was 5.78% in the low-dose rivaroxaban group and 6.45% in the standard-dose group.

**Figure 2:**
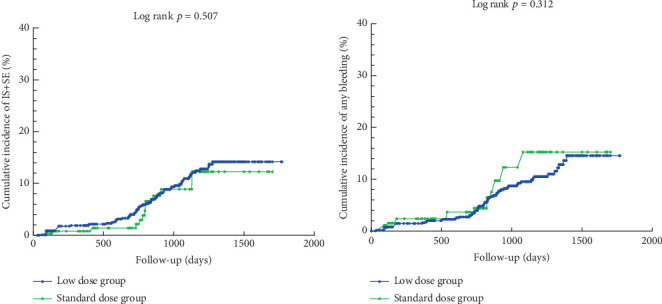
(a, b) Clinical outcomes of patients with different doses of rivaroxaban.

Figure 2 illustrates the cumulative incidence curves for IS+SE and any bleeding event in the low-dose and standard-dose rivaroxaban groups following adjustment using IPTW. The K–M analysis revealed no statistically significant difference in the risk of IS+SE between the low-dose and standard-dose groups (log-rank *p* = 0.507). Similarly, there was no significant difference in the risk of any bleeding event between the two groups (log-rank *p* = 0.312).

### 3.3. Risk Factors Associated With Efficacy and Safety Clinical Outcomes

**Figure 3:**
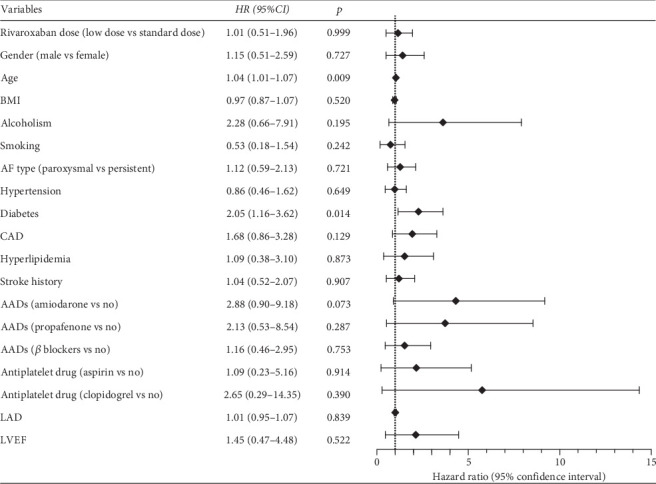
Independent influencing factors of IS+SE in patients with nonvalvular atrial fibrillation with normal renal function.

To exclude potential confounders, Cox regression analysis was conducted in the IPTW-adjusted population. As shown in Figure 3, after adjusting for demographic and clinical factors, patients in the low-dose group did not exhibit a statistically significant difference in the risk of IS+SE compared to those in the standard-dose group (HR = 1.01, 95% CI: 0.51–1.96, *p* = 0.999). However, diabetes (HR = 2.05, 95% CI: 1.16–3.62, *p* = 0.014) and older age (HR = 1.04, 95% CI: 1.01–1.07, *p* = 0.009) were associated with a higher risk of IS+SE. In detail, the risk of IS+SE in patients with diabetes was 2.05 times higher than in patients without diabetes. And for every year increase in age of NVAF patients, the risk of IS+SE increases by 1%.

**Figure 4:**
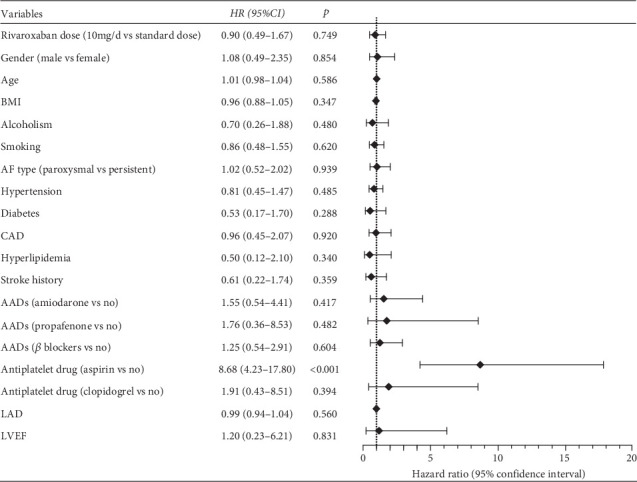
Independent influencing factors of any bleeding in patients with nonvalvular atrial fibrillation with normal renal function.

Similarly, Figure 4 demonstrates that a low dose of rivaroxaban did not significantly reduce the risk of any bleeding events compared to the standard dose (HR = 0.90, 95% CI: 0.49–1.67, *p* = 0.749). Notably, patients who also took aspirin were at a significantly higher risk of bleeding events (HR = 8.68, 95% CI: 4.23–17.80, *p* < 0.001).

### 3.4. Key Subgroup Analyses

**Figure 5:**
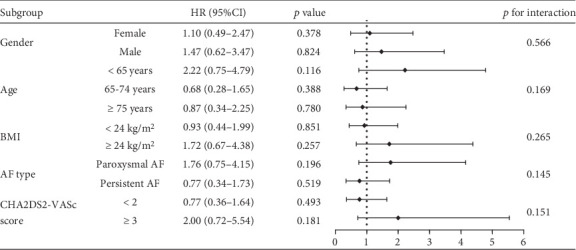
Subgroup analysis of the effect of low-dose rivaroxaban on the risk of IS+SE.

To evaluate the efficacy and safety of low-dose rivaroxaban in specific subgroups, we conducted subgroup analyses stratified by gender, age, BMI, AF type, CHA2DS2-VASc score, and HAS-BLED score.  Figure 5 illustrates the risk of IS+SE in different subgroups of NVAF patients treated with low-dose versus standard-dose rivaroxaban. The findings indicated no significant differences in the risk of IS+SE across all subgroups (all *p* > 0.05), indicating that the efficacy of low-dose rivaroxaban is comparable to that of the standard dose in preventing IS+SE, regardless of patient characteristics.

**Figure 6:**
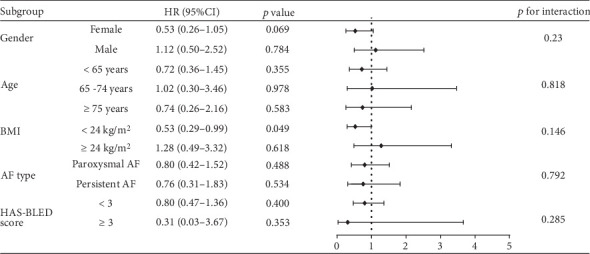
Subgroup analysis of the effect of low-dose rivaroxaban on the risk of any bleeding.

Figure 6 presents the subgroup analysis comparing the risk of bleeding between the low-dose and standard-dose groups. Notably, for patients with a BMI < 24 kg/m^2^, low-dose rivaroxaban was associated with a significantly reduced risk of bleeding (HR = 0.53, 95% CI: 0.29–0.99, *p* = 0.049). In contrast, for patients with a BMI ≥ 24 kg/m^2^, there was no significant difference in the risk of bleeding between the low-dose and standard-dose groups (HR = 1.28, 95% CI: 0.49–3.32, *p* = 0.618). Additionally, low-dose rivaroxaban did not significantly reduce the risk of bleeding when stratified by gender, age, AF type, or HAS-BLED score. This observation indicated that in clinical management, physicians should tailor the dosage of rivaroxaban according to the BMI of patients with NVAF to effectively minimize the risk of bleeding complications.

## 4. Discussion

In this study, we compared the efficacy and safety of low-dose rivaroxaban (10 mg/day) versus standard-dose rivaroxaban (15 or 20 mg/day) for stroke prevention in NVAF patients with a CrCl ≥ 50 mL/min. After excluding potential confounding factors, no significant differences were observed in the risk of IS+SE and any bleeding. Additionally, diabetes mellitus and older age were associated with a higher risk of IS+SE, while aspirin use was linked to increased bleeding risk. Subgroup analyses showed that in NVAF patients with a BMI < 24 kg/m^2^, low-dose rivaroxaban had a superior safety profile compared to the standard dose. In subgroups stratified by gender, age, AF type, and CHA2DS2-VASc scores, the efficacy of low-dose rivaroxaban was not inferior to that of the standard dose.

Substantial research has explored the efficacy and safety of low-dose rivaroxaban in NVAF patients. A 2018 Taiwanese study evaluated 6558 NVAF patients from the National Health Insurance Research Database (NHIRD) and found that low-dose rivaroxaban (10 mg/day) had comparable risks of thromboembolism and bleeding but an increased risk of myocardial infarction compared to standard doses (15 or 20 mg/day) [19]. However, the NHIRD lacked detailed renal function data, introducing ambiguity regarding appropriate dosing. A 2019 Japanese study addressed this by examining AF patients with a CrCl ≥ 50 mL/min, revealing that underdosed rivaroxaban was associated with higher risks of thromboembolic events and IS compared to standard doses [17]. These findings were also supported by a 2023 meta-analysis by Professor Gregory [22]. In contrast, a 2021 meta-analysis assessed Asian AF populations and found consistent risks of stroke and bleeding across different rivaroxaban doses (10, 15, or 20 mg/day) [23]. In the same year, Professor Shimokawa's study involving 6806 NVAF patients showed that for those with a CrCl ≥ 50 mL/min, a reduced dose of 10 mg/day did not significantly alter the risk of IS or SE compared to the standard dose of 15 mg/day [18]. Collectively, regional studies on the efficacy of 10 mg rivaroxaban in NVAF patients with a CrCl ≥ 50 have yielded inconsistent conclusions. Our research focused on mainland Chinese NVAF patients and demonstrated that low-dose (10 mg/day) rivaroxaban is a viable alternative to standard dose recommended in the ROCKET AF and J-ROCKET AF trials.

In real-world clinical practice, formulating antithrombotic strategies for patients with coexisting AF and CAD remains challenging. Physicians often worry that the concurrent use of antiplatelet and anticoagulant medications may increase the risk of bleeding. In our study, we observed a higher prevalence of CAD in the low-dose rivaroxaban group, highlighting the complexity of managing these comorbid conditions. Multiple studies have explored antithrombotic strategies for patients with AF and stable CAD, consistently concluding that monotherapy with rivaroxaban with a dose of 15 mg/day (10 mg/day for patients with a CrCl < 50 mL/min) is not inferior to combination therapy and may even be safer [24, 25]. Additionally, for patients with AF and acute coronary syndrome (ACS), a 2009 randomized double-blind clinical trial in the United States found that combining rivaroxaban (15 or 20 mg/day) with dual antiplatelet therapy (DAPT) significantly increased the risk of bleeding [26]. Similarly, other studies have shown that adding 10 mg/day of rivaroxaban to DAPT also elevates bleeding risk [27, 28]. Our study reached a similar conclusion: adding aspirin to anticoagulation therapy is associated with a higher risk of bleeding. A 2018 multicenter, randomized, double-blind cohort study published in the Lancet investigated the optimal dosage of combined aspirin and rivaroxaban therapy [29]. It revealed that for patients with CAD, treatment using aspirin plus rivaroxaban (2.5 mg twice daily) significantly reduced the risk of adverse cardiovascular events compared to aspirin monotherapy. And recent evidence has identified an optimal antithrombotic strategy for patients with ACS and AF. Adding very low-dose rivaroxaban (2.5 mg twice daily) to DAPT can lead to better clinical outcomes [30]. Consistent with this, current AF treatment guidelines recommend that for patients with AF and unstable coronary artery disease, if triple therapy (DAPT+rivaroxaban) is initiated, the dose of rivaroxaban should be 2.5 mg twice daily [31, 32].

Furthermore, age is a critical risk factor for both stroke and bleeding events [33, 34] and thus plays a pivotal role in guiding clinicians when formulating anticoagulation therapy plans for AF patients. Clinicians frequently opt for a reduced dose of rivaroxaban in older patients, primarily due to concerns about an increased risk of bleeding complications. This clinical practice is reflected in our patient cohort, where individuals in the low-dose rivaroxaban group are significantly older. Further subgroup analyses stratified by age demonstrate that, compared to the standard dose, low-dose rivaroxaban exhibited equivalent efficacy in preventing strokes, despite not reducing the risk of bleeding. This suggests that the reduced dose of rivaroxaban maintains its therapeutic effectiveness for stroke prevention without compromising safety in terms of bleeding events. A 2023 systematic review proposed that lower doses of direct oral anticoagulants (DOACs) may be associated with reduced mortality in elderly AF patients compared to standard doses [35]. Additionally, a 2023 Chinese study indicated that 10 mg once-daily rivaroxaban may provide survival benefits for elderly patients with NVAF [36]. These findings offer important insights for clinical practice, supporting the use of low-dose rivaroxaban as a viable and potentially superior option for anticoagulation therapy in older patients, where balancing efficacy and safety is crucial. The evidence underscores the importance of individualized dosing strategies, particularly for elderly patients, to optimize both therapeutic outcomes and patient safety.

Otherwise, our study found that diabetes was associated with a higher risk of IS+SE, which was aligned with previous findings. Recent research by Chinese scholars this year confirmed that diabetes was an independent risk factor for SE, mortality, and cardiovascular events in AF patients [37]. Given that diabetes is also a component of the CHA2DS2-VASc score, it underscores the importance of emphasizing anticoagulation therapy in AF patients with diabetes.

Notably, the subgroup analysis of this study demonstrated that low-dose rivaroxaban significantly reduces the risk of bleeding events in patients with a BMI of less than 24 kg/m^2^ while maintaining comparable efficacy in stroke prevention. This finding is consistent with previous studies. A nationwide cohort study conducted in the United Kingdom in 2022 investigated the risks and benefits of oral anticoagulants in NVAF patients across different BMI categories. The final results indicated that underweight patients (BMI < 18.5 kg/m^2^) exhibited distinct risk-benefit patterns with oral anticoagulation compared to other BMI categories. Specifically, these patients may face a higher risk of bleeding events [38]. Additionally, a study by Italian scholar Giuseppe Patti, which included 9330 participants, also demonstrated that underweight patients (BMI < 24.6 kg/m^2^) should be considered for optimized anticoagulation strategies to ensure maximum safety, given their higher risk of bleeding [39]. Low body weight patients tend to have an increased risk of bleeding when treated with NOACs, potentially due to variations in drug distribution, clearance, and reduced coagulation factor levels [40, 41]. These findings collectively highlight the necessity of appropriate dose reduction in anticoagulant therapy for nonoverweight patients (BMI < 24 kg/m^2^) with NVAF. To balance thrombotic protection and bleeding prevention, it is essential to adopt individualized dosing strategies that consider patient-specific factors such as BMI. This approach aligns with current clinical guidelines and supports the optimization of anticoagulation therapy to achieve the best possible outcomes for NVAF patients.

This study has several limitations. Firstly, it is a single-center, observational retrospective study with a relatively small sample size. Secondly, dosing information for rivaroxaban was obtained from medical records and telephone follow-ups, potentially introducing information bias. Thirdly, patient loss to follow-up might lead to an underestimation of event rates. Fourthly, the study is based solely on the initial dose of rivaroxaban and baseline patient characteristics; it does not account for any changes in dose or CrCl levels during the study period, nor does it consider invasive treatments such as catheter ablation or surgery.

## 5. Conclusion

In this observational, retrospective study, we demonstrated that low-dose rivaroxaban (10 mg/day) is a viable alternative to standard doses for stroke prevention in Chinese NVAF patients with a CrCl ≥ 50 mL/min, especially in nonoverweight individuals where it reduces bleeding risk without compromising efficacy.

## Figures and Tables

**Table 1:** Comparison of baseline characteristics of patients before inverse probability weighting.

	**Total (** **n** = 1455**)**	**Low dose group (** **n** = 1176**)**	**Standard dose group (** **n** = 279**)**	**p**
Female (%)	814 (55.95)	654 (55.61)	160 (57.35)	0.647
Age (mean ± *SD*) (years)	66.98 ± 11.16	68.00 ± 10.67	62.67 ± 12.13	< 0.001
BMI (mean ± *SD*) (kg/m^2^)	23.67 ± 3.14	23.60 ± 3.13	23.95 ± 3.20	0.101
Alcoholism (%)	171 (11.75)	130 (11.05)	41 (14.69)	0.111
Smoking (%)	269 (18.49)	211 (17.94)	58 (20.79)	0.310
CHA2DS2-VASc score ≥ 2 (%)	774 (53.20)	632 (53.74)	142 (50.89)	0.417
HAS-BLED score ≥ 3 (%)	44 (3.02)	34 (2.89)	10 (3.58)	0.679
AF type (%)				0.003
Paroxysmal AF	814 (55.95)	635 (54.00)	179 (64.16)	
Persistent AF	641 (44.05)	541 (46.00)	100 (35.84)	
Hypertension (%)	568 (39.04)	459 (39.03)	109 (39.07)	0.998
Diabetes (%)	228 (15.67)	180 (15.31)	48 (17.20)	0.489
CAD (%)	492 (33.81)	428 (36.39)	64 (22.94)	< 0.001
Hyperlipidemia (%)	69 (4.74)	50 (4.25)	19 (6.81)	0.099
Stroke history (%)	286 (19.66)	235 (19.98)	51 (18.28)	0.625
AADs (%)				0.401
No	57 (3.92)	47 (3.99)	10 (3.58)	
Amiodarone	253 (17.39)	196 (16.67)	57 (20.43)	
Propafenone	49 (3.37)	39 (3.32)	10 (3.58)	
*β*-Blockers	1096 (75.32)	894 (76.02)	202 (72.41)	
Antiplatelet drug (%)				0.586
No	1387 (95.33)	1123 (95.49)	264 (94.62)	
Aspirin	54 (3.71)	41 (3.49)	13 (4.66)	
Clopidogrel	14 (0.96)	12 (1.02)	2 (0.72)	
LAD (mean ± *SD*) (mm)	40.24 ± 7.65	40.33 ± 7.66	39.95 ± 7.56	0.454
LVEF (mean ± *SD*) (%)	63.01 ± 17.55	63.20 ± 19.51	61.83 ± 7.73	0.539
Follow-up time (quantile)	734 (180, 1115)	743.5 (180, 1126.75)	705 (180, 940)	0.211

**Table 2:** Comparison of baseline characteristics of patients after inverse probability weighting.

	**Rivaroxaban (low dose) (** **w** **e** **i** **g** **h** **t** **sample** **size** = 1454.3**)**	**Rivaroxaban (standard dose) (** **w** **e** **i** **g** **h** **t** **sample** **size** = 1465.6**)**	**p**	**SMD**
Female (%)	810.3 (55.72)	771.9 (52.67)	0.421	0.061
Age (mean ± *SD*)	66.99 ± 11.10	67.87 ± 12.87	0.318	0.083
BMI (mean ± *SD*)	23.65 ± 3.13	23.41 ± 3.17	0.788	0.020
Alcoholism (%)	171.8 (11.81)	171.6 (11.71)	0.961	0.003
Smoking (%)	268.8 (18.48)	254.5 (17.36)	0.674	0.029
CHA2DS2-VASc score ≥ 2 (%)	745.8 (51.28)	735.9 (50.21)	0.777	0.021
HAS-BLED score ≥ 3 (%)	43.6 (3.00)	43.2 (2.95)	0.964	0.003
AF type (%)			0.853	0.014
Paroxysmal AF	813.7 (55.95)	830.4 (56.66)		
Persistent AF	640.6 (44.05)	635.2 (43.34)		
Hypertension (%)	566.0 (38.92)	533.9 (36.43)	0.492	0.051
Diabetes (%)	227.9 (15.67)	216.2 (14.75)	0.712	0.026
CAD (%)	492.0 (33.83)	447.5 (30.53)	0.371	0.071
Hyperlipidemia (%)	68.4 (4.70)	63.1 (4.31)	0.754	0.019
Stroke history (%)	285.5 (19.63)	274.7 (18.74)	0.783	0.023
AADs (%)			0.263	0.157
No	117.8 (8.10)	67.6 (4.61)		
Amiodarone	184.4 (12.68)	222.0 (15.15)		
Propafenone	47.8 (3.29)	42.0 (2.87)		
*β*-Blockers	1104.3 (75.93)	1134.0 (77.37)		
Antiplatelet drug (%)			0.510	0.071
No	1389.1 (95.52)	1414.2 (96.49)		
Aspirin	50.3 (3.46)	44.7 (3.05)		
Clopidogrel	14.9 (1.02)	6.7 (0.46)		
LAD (mean ± *SD*)	40.27 ± 7.66	40.58 ± 8.47	0.656	0.038
LVEF (mean ± *SD*)	63.41 ± 18.14	62.34 ± 7.02	0.235	0.059
Follow-up time (quantile)	749 (154, 1236)	728 (139, 1307)	0.812	0.014

## Data Availability

The dataset analyzed during the current study is not publicly available due to a lack of consent from study participants to do so, but it is available from the corresponding authors on reasonable request for researchers who meet the criteria for access to confidential data.
